# Detection of phosphates originating from Enceladus’s ocean

**DOI:** 10.1038/s41586-023-05987-9

**Published:** 2023-06-14

**Authors:** Frank Postberg, Yasuhito Sekine, Fabian Klenner, Christopher R. Glein, Zenghui Zou, Bernd Abel, Kento Furuya, Jon K. Hillier, Nozair Khawaja, Sascha Kempf, Lenz Noelle, Takuya Saito, Juergen Schmidt, Takazo Shibuya, Ralf Srama, Shuya Tan

**Affiliations:** 1 Institut für Geologische Wissenschaften, Freie Universität Berlin, Berlin, Germany; 2 Earth-Life Science Institute (ELSI), Tokyo Institute of Technology, Tokyo, Japan; 3 Institute of Nature and Environmental Technology, Kanazawa University, Ishikawa, Japan; 4 Space Science Division, Space Sector, Southwest Research Institute, San Antonio, TX USA; 5 Leibniz-Institute für Oberflächenmodifizierung, Leipzig, Germany; 6 Institute of Chemical Technology, University of Leipzig, Leipzig, Germany; 7 Laboratory for Atmospheric and Space Physics (LASP), University of Colorado, Boulder, CO USA; 8 Institute for Extra-cutting-edge Science and Technology Avantgarde Research (X-star), Japan Agency for Marine-Earth Science and Technology (JAMSTEC), Kanagawa, Japan; 9 Astronomy Research Unit, University of Oulu, Oulu, Finland; 10 Institut für Raumfahrtsysteme, Universität Stuttgart, Stuttgart, Germany

**Keywords:** Rings and moons, Geochemistry

## Abstract

Saturn’s moon Enceladus harbours a global^1^ ice-covered water ocean^2,3^. The Cassini spacecraft investigated the composition of the ocean by analysis of material ejected into space by the moon’s cryovolcanic plume^4–9^. The analysis of salt-rich ice grains by Cassini’s Cosmic Dust Analyzer^10^ enabled inference of major solutes in the ocean water (Na^+^, K^+^, Cl^–^, HCO_3_^–^, CO_3_^2–^) and its alkaline pH^3,11^. Phosphorus, the least abundant of the bio-essential elements^12–14^, has not yet been detected in an ocean beyond Earth. Earlier geochemical modelling studies suggest that phosphate might be scarce in the ocean of Enceladus and other icy ocean worlds^15,16^. However, more recent modelling of mineral solubilities in Enceladus’s ocean indicates that phosphate could be relatively abundant^17^. Here we present Cassini’s Cosmic Dust Analyzer mass spectra of ice grains emitted by Enceladus that show the presence of sodium phosphates. Our observational results, together with laboratory analogue experiments, suggest that phosphorus is readily available in Enceladus’s ocean in the form of orthophosphates, with phosphorus concentrations at least 100-fold higher in the moon’s plume-forming ocean waters than in Earth’s oceans. Furthermore, geochemical experiments and modelling demonstrate that such high phosphate abundances could be achieved in Enceladus and possibly in other icy ocean worlds beyond the primordial CO_2_ snowline, either at the cold seafloor or in hydrothermal environments with moderate temperatures. In both cases the main driver is probably the higher solubility of calcium phosphate minerals compared with calcium carbonate in moderately alkaline solutions rich in carbonate or bicarbonate ions.

## Abstract (web summary)

Cassini’s Cosmic Dust Analyzer mass spectra of ice grains emitted by Enceladus show the presence of sodium phosphates, suggesting that phosphorus is readily available in Enceladus’s ocean in the form of orthophosphates.

## Main

Enceladus’s global ocean lies under an ice crust and above a rocky core where tidal dissipation^18,19^ is suspected to drive hydrothermal activity^4,5^. There are several lines of evidence describing how volatile^5^ and dissolved materials^3,4,11^ from the rocky core are either emitted by the plume in the gas phase or incorporated into ice particles, respectively.

Cassini’s Cosmic Dust Analyzer (CDA) recorded time-of-flight (ToF) mass spectra with a mass resolution of *m*/Δ*m* ≈ 10–50 (refs. ^10,20^) for cations generated by high-velocity impacts of individual grains onto the instrument’s rhodium target. The E-ring of Saturn is formed by ice grains escaping Enceladus’s plume into orbits around Saturn^21^, and hence the analysis of these grains by CDA provides important insights into the composition of the subsurface ocean—including a rich variety of organic compounds^6–9^—with much better statistics compared with data from the rare occasions when Cassini traversed the plume itself.

In a previous analysis it was shown that a fraction of these ice grains, called Type 3 particles, contain salts at significantly higher concentrations than all other ice grains in the plume and the E-ring^3,11^. By co-addition of spectra from 107 individual detections in the E-ring, average salt concentrations of 0.5–2.0% by weight (around 0.07–0.30 M) have been deduced for Type 3 grains, with NaCl, NaHCO_3_, Na_2_CO_3_ and KCl as the most abundant constituents. Because it was inferred that these grains are derived from aerosolized ocean water^3^, the salt chemistry of the plume and E-ring grains would reflect that of the ocean.

Phosphorus is an element essential for planetary habitability^12–14^, but to date it has not been detected in an ocean beyond Earth. Previous geochemical modelling suggested that phosphate might be scarce in the ocean of Enceladus and other icy ocean worlds^15,16^. However, more recent modelling of mineral solubilities in Enceladus’s ocean suggests that phosphate could be relatively abundant (roughly 10^−7^–10^−2^ M), depending on the compositional characteristics of the ocean such as its pH and carbonate content, as well as on attainment of certain chemical equilibria between ocean water and seafloor alteration minerals^17^.

In this work we present CDA mass spectra of a population of E-ring ice grains that show the presence of sodium phosphates. We then perform laboratory analogue experiments to quantitatively establish that Enceladus’s ocean is rich in dissolved phosphate. Lastly, we show through water–rock alteration experiments and complementary chemical speciation calculations that phosphate-rich fluids are an inevitable outcome of alkaline and carbonate-rich ocean water reacting with unaltered carbonaceous chondritic rock.

## Results

**Fig. 1:**
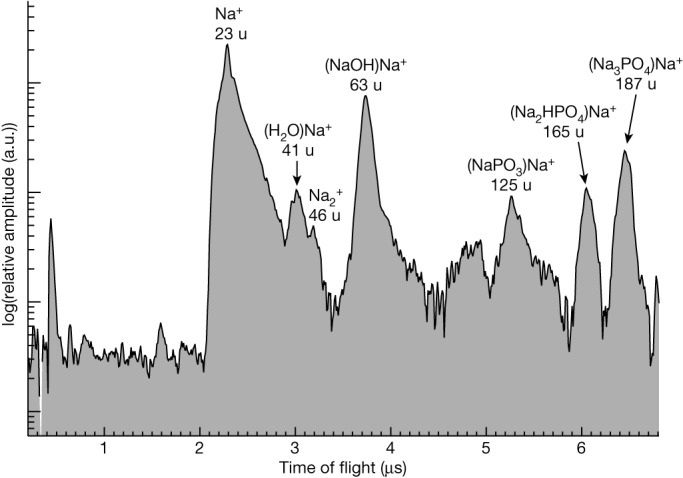
CDA cation spectrum co-added from nine baseline-corrected individual ice grain spectra. The mass lines signifying a high-salinity Type 3 spectrum are Na^+^ (23 u) and (NaOH)Na^+^ (63 u) with secondary Na-rich signatures of (H_2_O)Na^+^ (41 u) and Na_2_^+^ (46 u). Sodium phosphates are represented by phosphate-bearing Na-cluster cations, with (Na_3_PO_4_)Na^+^ (187 u) possessing the highest amplitude in each spectrum followed by (Na_2_HPO_4_)Na^+^ (165 u) and (NaPO_3_)Na^+^ (125 u). The first two unlabelled peaks at the beginning of the spectrum are H^+^ and C^+^, stemming from target contamination^39^. a.u., arbitrary units.

We conducted a comprehensive survey of 345 Type 3 particles (Methods, Extended Data Table 1). Our analysis led to the detection of a rare subtype (nine particles; Methods, Extended Data Table 2) whose spectra show highly significant features of phosphate species: in addition to the defining mass spectral peaks of Na-salt-rich water ice grains at 23 and 63 u (ref. ^3^), there were peaks corresponding to molecular masses of 125, 165 and 187 u forming a pattern we found to be unique to sodium phosphates at high concentrations within these ice grains (Fig. 1 and Methods).

**Fig. 2:**
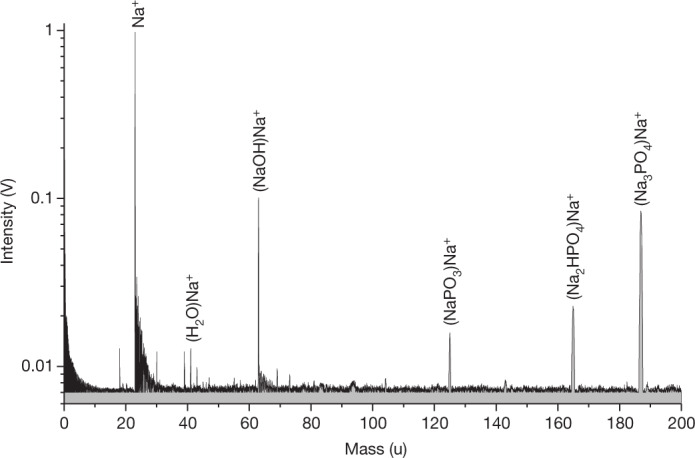
Spectrum from the LILBID analogue experiment reproducing the features in the CDA spectrum. An aqueous solution of 0.420 M Na_2_HPO_4_ and 0.038 M Na_3_PO_4_ was used. All major characteristics of the CDA spectrum of phosphate-rich grains (Fig. 1) are reproduced at the higher mass resolution of the laboratory mass spectrometer (roughly 700 *m*/Δ*m*). Note: this solution is not equivalent to the inferred ocean concentration. To derive the latter quantity, the concentration determined in these P-rich grains must be averaged over the entire dataset of salt-rich ice grains (see below).

**Extended Data Fig. 1:**
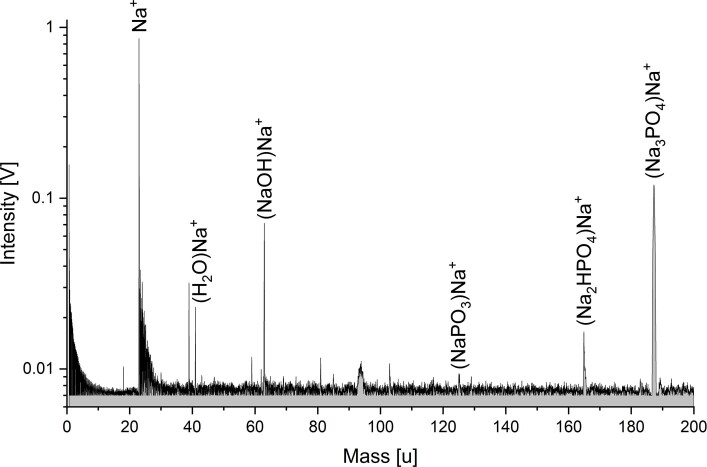
LILBID spectrum of a 0.1 M solution of Na_3_PO_4_. The (NaPO_3_)Na^+^ peak (125 u) cannot be reproduced with sufficient amplitude and the ratio of (Na_3_PO_4_)Na^+^ (187 u) to (Na_2_HPO_4_)Na^+^ (165 u) is too high compared with CDA spectra. Both these disagreements disappear if sufficient amounts of Na_2_HPO_4_ are added to the solution.

**Extended Data Fig. 2:**
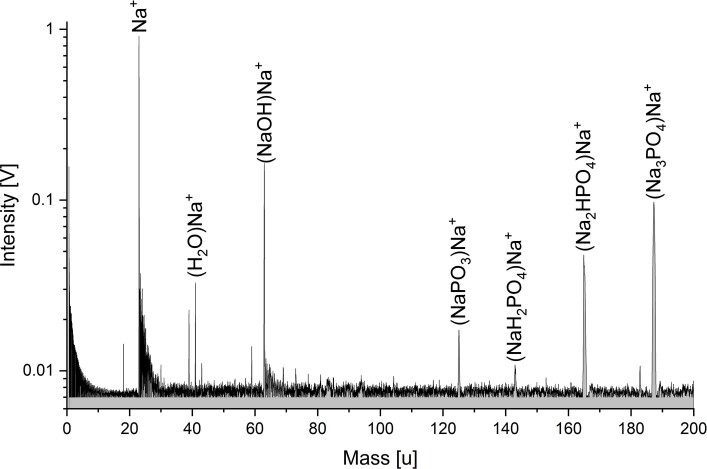
LILBID spectrum of a 0.1 M solution of Na_2_HPO_4_. A mass line at 143 u ((NaH_2_PO_4_)Na^+^) appears, which is not apparent in CDA spectra except tentatively in spectrum #3 of Extended Data Table 2. This signature at 143 u is not present in a mixture of Na_2_HPO_4_ and Na_3_PO_4_ (Fig. 2). This motivates our conclusion on a mixture of the two orthophosphates in the ice grains from Enceladus analysed by CDA.

To obtain a quantitative assessment of the composition of these phosphate-bearing ice grains, an analogue experiment was used to simulate the impact ionization of ice grains via a laser pulse targeting a micrometre-sized water beam^22^. This technique, laser-induced desorption of ions and ionic aggregates^23^, has previously reproduced accurate CDA ice grain spectra^3,9^. Despite the higher amplitude of the (Na_3_PO_4_)Na^+^ peak compared with that of (Na_2_HPO_4_)Na^+^, the peak pattern can best be reproduced by dissolving Na_3_PO_4_ and Na_2_HPO_4_ at an inverse salt molar mixing ratio between the two salts of 1:2.5–1:25 (Fig. 2), indicating the dominance of monohydrogen phosphate (Na_2_HPO_4_) over phosphate (Na_3_PO_4_) in these ice grains. The dominance of Na_2_HPO_4_ is substantiated by the presence of (NaPO_3_)Na^+^ (125 u), a charged molecular meta phosphate aggregate (Methods, Extended Data Fig. 4) that formed from Na_2_HPO_4_ in our analogue experiments, but not from Na_3_PO_4_ alone (Methods, Extended Data Figs. 1 and 2). With the molar ratio of Na_2_HPO_4_:Na_3_PO_4_ kept above 2.5:1.0, a concentration ranging from 0.05 to 0.60 M of total phosphate salts then gave a close match with CDA spectra of phosphate-rich grains (Methods).

**Extended Data Fig. 3:**
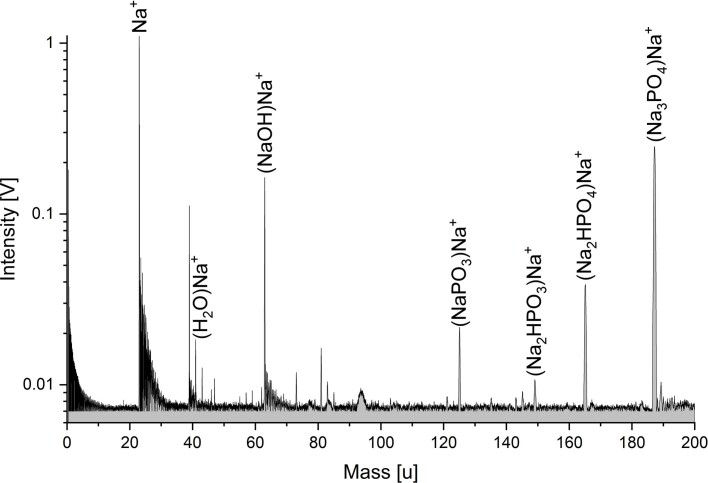
LILBID spectrum with a 0.5 M phosphate concentration (similar to Fig. 2) and 0.01 M phosphite (Na_2_HPO_3_). The phosphite peak Na_2_HPO_3_Na^+^ (149 u) is not in agreement with CDA spectra and only becomes undetectable in phosphate-bearing CDA spectra at phosphite concentrations below 0.005 M. Note, the CDA detection limit of phosphite in pure water (without phosphates) is approx. 1 nM, more than 3 orders of magnitude lower.

We also tested for the presence of phosphites (for example, Na_2_HPO_3_, where P has an oxidation state of +III compared with +V in the orthophosphates Na_3_PO_4_ and Na_2_HPO_4_). Because the acid dissociation constant (pK_a_) of H_2_PO_3_^–^ is about 6.2 at 0 °C (ref. ^24^), this species is unlikely to be relevant to Enceladus’s alkaline ocean^3,11^. Raising the concentration of Na_2_HPO_3_ in the phosphate mixture to 5 mM and above led to a clear mass line at 149 u due to (Na_2_HPO_3_)Na^+^ (Methods and Extended Data Fig. 3). However, this peak was not observed in CDA spectra (5.7–5.8 µs in Fig. 1), thus providing an upper limit on phosphite concentrations in these ice grains that is one to two orders of magnitude below the required phosphate concentration.

We now follow the approach established in Postberg et al.^3^, which assumes that the total ensemble of salt-rich grains emitted currently by the plume is the best representation of the composition of the source water. Thus, to infer the concentration in the ocean we calculate the average phosphate concentration in all Type 3 grains. We ‘dilute’ the concentration in the nine spectra of the new phosphate-bearing subtype (2.6 ± 0.9% by number) with the 336 spectra of other Type 3 grains—which are mostly dominated by NaCl or Na_2_CO_3_ (ref. ^3^)—and do not show phosphates at detectable concentrations ([ΣPO_4_^3–^] < 5 µM). Including the ±0.9% s.e.m. from counting statistics and the inferred concentrations of total dissolved phosphate ([ΣPO_4_^3–^] = 0.05 – 0.60 M; [ΣPO_4_^3–^] = [PO_4_^3–^] + [HPO_4_^2–^] + [H_2_PO_4_^–^], where [*X*] denotes the molar concentration of species *X*), this yields a phosphate concentration range of 0.8–21 mM for the Enceladean ocean. From our analogue experiments, the upper limit inferred from the nondetection of phosphites lies at least one order of magnitude below phosphate concentrations. This indicates a clear predominance of orthophosphates as the most abundant P-bearing species in the Enceladean ocean.

**Fig. 3:**
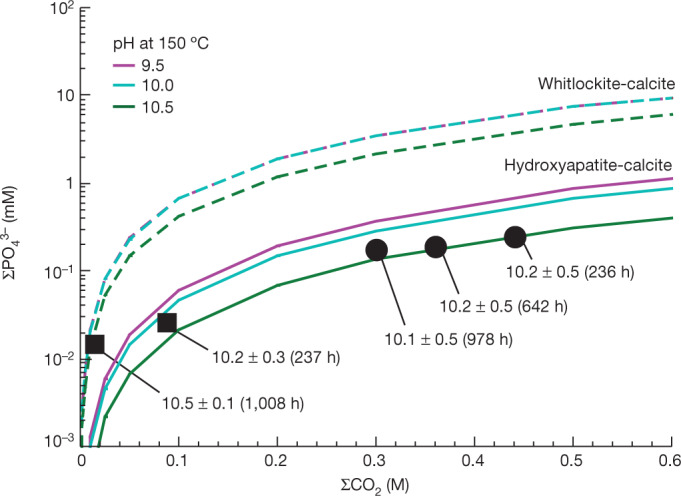
Phosphate concentrations derived from hydrothermal experiments. Experimental results (squares for Run no. 1 and circles for Run no. 2, see text) of P concentrations in hydrothermal fluids at 150 °C and 30 MPa are shown as a function of ΣCO_2_ concentration. Numbers attached to data points are calculated in situ pH (Methods) and reaction times in hours. Solid and dashed curves represent results from thermodynamic equilibrium calculations (Methods) for hydroxyapatite-calcite and whitlockite-calcite buffer systems, respectively, evaluated at different pH values.

To interpret the CDA detection of phosphates we performed hydrothermal laboratory experiments with powdered samples from a carbonaceous chondrite (Jbilet Winselwan (CM-2 type))^25^ as an analogue of Enceladus’s core material^26^. The main purpose of the hydrothermal experiments was to elucidate the geochemical controls on phosphate solubility under conditions relevant to Enceladus. The chondritic material was exposed to two different Na-carbonate-enriched solutions (Run no. 1: initial ΣCO_2_ = 0.2 M; Run no. 2: initial ΣCO_2_ = 1.0 M), where the total dissolved inorganic carbon concentration is given by ΣCO_2_ = [CO_2,aq_] + [HCO_3_^–^] + [CO_3_^2–^] + [NaCO_3_^–^] + [NaHCO_3_]. The starting solution also contained 0.5 M ΣNH_3_ = [NH_3,aq_] + [NH_4_^+^], a plausible concentration for Enceladus’s ocean^5^. An elevated temperature (150 °C) was used in the experiment to accelerate reaction rates. This allowed us to study key aspects of the water–rock interactions occurring in Enceladus at time scales accessible in the laboratory of up to about 1,000 h. Fluid samples were collected periodically from the reaction cell during the experiments to determine pH and fluid composition, including the concentrations of P and ΣCO_2_ (Methods). The initial [ΣCO_2_] adopted for Run no. 2 (1.0 M) was higher than the value inferred for the Enceladean ocean^27,28^, but a large fraction of initial ΣCO_2_ was removed during the experiments via formation of carbonate minerals (Methods), leading to more realistic levels of carbonate species (Fig. 3).

Our experiments show that the dissolved phosphate concentration increases with in situ measured ΣCO_2_ (Fig. 3). This happens because an increase in CO_3_^2–^ concentration (or, more correctly, aqueous activity) leads to dissolution of Ca phosphate minerals with the formation of stable Ca carbonates (that is, calcite)^17,29,30^. In comparison with thermodynamic equilibrium predictions, we find that the hydroxyapatite-calcite buffer system (hydroxyapatite + 5CO_3_^2–^ + H^+^ ↔ calcite + 3PO_4_^3–^ + H_2_O) is generally applicable to our experimental results (Fig. 3). The relevance of this buffer is further supported by chemical analysis of the solid residues after Run no. 2, which demonstrates the presence of coexisting Ca phosphate with high Ca:P ratios (probably a mixture of hydroxyapatite and Ca-/Mg-whitlockite) and calcite (Methods, Extended Data Figs. 5–7, Extended Data Table 4). One data point in Fig. 3 (ΣCO_2_ = 0.01 M in Run no. 1) lies significantly above the hydroxyapatite-calcite buffer, appearing to be more consistent with the whitlockite-calcite buffer (whitlockite (β-Ca_3_(PO_4_)_2_) + 3CO_3_^2–^ ↔ calcite + 2PO_4_^3–^). We suspect that this change in behaviour is due to unusually high concentrations of Ca^2+^ and Mg^2+^ in Run no. 1 (0.8 and 0.3 mM, respectively; Methods, Extended Data Fig. 8, Extended Data Table 3), which may promote the precipitation of whitlockite^31^. This interpretation is supported by an electron probe microanalysis of the solid residue (Methods, Extended Data Table 4). Alternatively the CM chondrite, Jbilet Winselwan (Extended Data Figs. 9 and 10), used in our experiments is known to contain impact-induced, thermally metamorphosed materials^25^, which could make some of the results less relevant to Enceladus. By contrast, buffer systems involving other phosphates (for example, Mg-whitlockite and merrillite) and carbonates (for example, dolomite) cannot explain the experimental results (Methods).

**Fig. 4:**
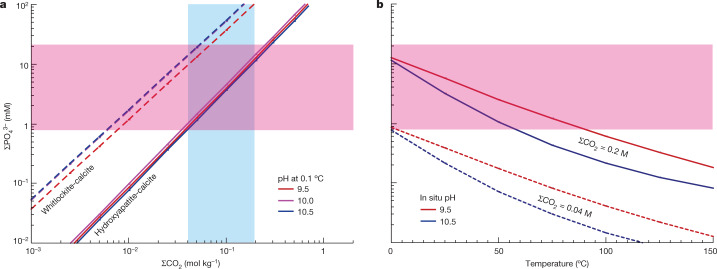
Comparison of observed and calculated concentrations of ΣPO_4_^3–^ in fluids affected by water–rock reactions within Enceladus. **a**, Relation between ΣPO_4_^3–^ and ΣCO_2_ at a temperature of 0.1 °C for the hydroxyapatite-calcite buffer system (solid lines) and the whitlockite-calcite buffer system (dashed lines). Constraints on ΣCO_2_ obtained in previous studies^27,28^ are indicated by the blue shaded area. The area highlighted in pink represents the range of ΣPO_4_^3–^ constrained in this study from CDA data. **b**, Dependence of ΣPO_4_^3–^ on temperature for the hydroxyapatite-calcite buffer and different values of pH and ΣCO_2_. A similar diagram for the whitlockite-calcite buffer can be found in Extended Data Fig. 11.

Figure 4a shows the modelled ΣPO_4_^3–^ as a function of ΣCO_2_ for water–rock interactions adopting the two buffer systems inferred from our high-temperature experiments, but now transferred to conditions corresponding to the cold seafloor of Enceladus (0.1 °C and 8 MPa). The P concentrations determined from the CDA measurements in this work (ΣPO_4_^3–^ = 0.8–21 mM), the independently derived constraint on ΣCO_2_ (0.04–0.20 M)^27,28^, as well as an alkaline pH (9.0–10.5)^3,4^, are all fully consistent with these two buffer systems. The hydroxyapatite-calcite buffer is applicable for the entire range of ΣCO_2_ (Fig. 4a), corresponding to ΣPO_4_^3–^ of about 0.8–12 mM. The whitlockite-calcite buffer is applicable for low ΣCO_2_ of about 0.04–0.07 M corresponding to ΣPO_4_^3–^ in the range of about 7–21 mM at 0.1 °C.

Figure 4b shows the temperature dependence of ΣPO_4_^3–^ in fluids controlled by the hydroxyapatite-calcite buffer. The effects of colder fluids, as used in the experiments, down to oceanic temperatures near 0 °C can be seen. The phosphate concentration increases with lower temperature at constant pH because hydroxyapatite (and other reactants) becomes less stable relative to calcite (and other products) at lower temperatures. To achieve ΣPO_4_^3–^ in the range 0.8–21 mM, as constrained by the CDA measurements, the temperature of the water–rock reactions that provide phosphate in Enceladus would need to be 80 °C or less if the pH is 9.5, and below 65 °C if the pH is 10.5 (Fig. 4b). Higher-temperature fluids could contain sufficient phosphate only if the fluid pH were lower. If the whitlockite-calcite buffer controls ΣPO_4_^3–^ in Enceladus’s ocean, the temperature could be as high as around 180 °C (Extended Data Fig. 11) for a maximum ΣCO_2_ of 0.2 M. In the case in which high-temperature interactions with seafloor rocks provide the oceanic phosphate, its concentration in the fluids should be preserved on cooling to near subzero temperature within the cold ocean. This scenario would, however, be relevant only if the time scale of cycling the ocean through hydrothermal systems is shorter than that of low-temperature dissolution of phosphate minerals at the seafloor.

Given the consistency of the measured ΣPO_4_^3–^, ΣCO_2_ and pH of Enceladus’s ocean, we suggest that thermodynamic equilibrium between Ca phosphate (for example, hydroxyapatite) and calcite is probably achieved in both the cold global ocean and hydrothermal fluids of moderate temperature.

## Discussion

The CDA detection of ice grains with high concentrations of orthophosphates indicates that phosphorus is readily available at the top of Enceladus’s ocean (that is, the plume source region). Even with a conservative margin, our estimate indicates concentrations in the order of at least hundreds of micromolar, several 100-fold the average phosphate abundance in Earth’s oceans^32^. The heterogenous distribution of salts in the emitted ice grains might suggest some level of heterogeneity in the ocean and that actual concentrations could be locally higher or lower than the bulk number calculated in this work. Alternatively, yet unknown mechanisms might separate different salts when ocean material is transported upwards in the vents. However, even without knowing the details of the subsurface processes, we assume that the total ensemble of salt-rich grains emitted by the plume is a reasonable representation of the average ocean composition near its surface.

A phosphate:phosphite ratio of at least around 10:1, as implied by our results, is consistent with the much greater thermodynamic stability of phosphates relative to phosphites under Enceladean ocean conditions^17^. In addition, the nondetection of phosphites suggests that aqueous alteration of iron phosphides (the most probable accreted form of P) is no longer a significant source of P for Enceladus’s ocean. This process would produce metastable phosphites^33^. The inferred lack of contemporary alteration of phosphides is consistent with the presence of a low-density rocky core that has already experienced extensive water–rock interaction^2,18,34,35^. Phosphites could have been produced when accreted phosphides first reacted with liquid water, but then might have been cycled through hydrothermal systems where they would have been converted to more stable phosphates.

The time scale of our experiments is much shorter than those of water–rock reactions in Enceladus, giving rise to uncertainties in the exact phosphate/carbonate minerology inside the icy moon. Recent work^17^ estimated [ΣPO_4_^3–^] in Enceladus’s ocean at low temperatures, as achieved by interactions involving thermodynamically stable phosphate and carbonate minerals (for example, merrillite and dolomite). The upper end of the predicted range from that study also agrees with the ocean concentration of phosphates (0.8–21 mM) derived here if the ocean pH is 8.0–10.5. This is consistent with the range of 9.0–10.5 adopted in this work. A notable difference is that the range given by ref. ^17^ also permitted [ΣPO_4_^3–^] to reach much lower values—specifically at pH above 10.0—than those based on the hydroxyapatite-calcite buffer in Fig. 4a. The probable reason for this is that, under the strongly carbonated conditions modelled by ref. ^17^, hydroxyapatite was more soluble and thus merrillite took over the role of the P-controlling phosphate mineral. It is important to note that both systems produce results in good agreement with the CDA measurements and, in both cases, the driver enabling the abundant availability of phosphate is the high observed concentration of dissolved carbonate species, which shift phosphate-carbonate mineral equilibria toward dissolution of solid phosphates into Enceladus’s ocean.

An earlier study by Lingam and Loeb^15^ suggested that the availability of phosphorus would be the bottleneck of bio-essential elements on Enceladus and other icy ocean worlds with potential hydrothermal activity and without dry land. Steady-state concentrations “likely lower than the corresponding value on Earth by a few orders of magnitude”^15^ would markedly reduce the prospects for life. Indeed, of the six elements—C, H, N, O, P and S—that are generally considered to be critical ingredients for life based on water and organic chemistry^12^, phosphorus is cosmochemically the least abundant and has not previously been detected at any of the ocean-bearing moons in the Solar System. However, the results presented here demonstrate that Enceladus instead has a high availability of dissolved P, which is thus extremely unlikely to be a limiting factor in the survival of putative life on Enceladus—and perhaps also on other ocean worlds that reside beyond the CO_2_ snowline in the Solar System (that is, the distance from the Sun beyond which CO_2_ is in a solid (icy) state and is available as a planetary building material).

**Fig. 5:**
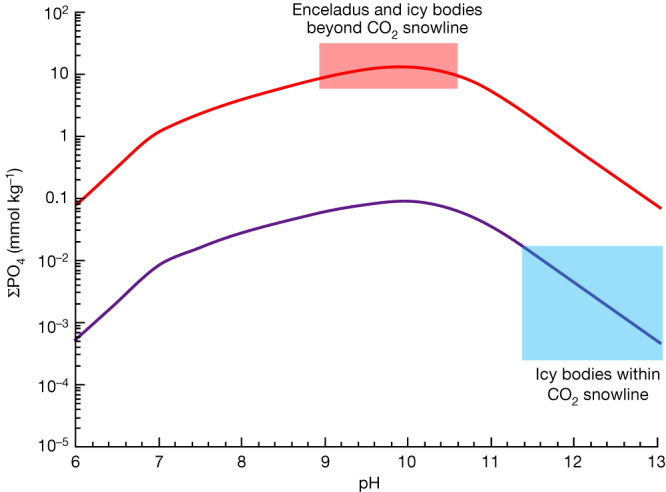
Sensitivity of ΣPO_4_^3–^ of the hydroxyapatite-calcite buffer system to pH and ΣCO_2_ at 0.1 °C and 8 MPa. The case of ΣCO_2_ = 0.2 M (red curve) corresponds to the ocean water of Enceladus^3^ and, perhaps, to icy bodies beyond the CO_2_ snowline that originally contained CO_2_ ice. The typical pH of such ΣCO_2_-rich seawater is around 9.0–11.0 at W:R of approximately 1.0, owing to high concentrations of dissolved carbonates^16,36^. The case of ΣCO_2_ = 0.01 M (purple curve) might correspond to ocean water of icy bodies within the CO_2_ snowline that originally contained no CO_2_ ice. According to ref. ^16^, ΣCO_2_ in fluid becomes in the order of 0.01 M by interactions between CI chondritic rocks and pure water at 25 °C, around 1 bar and W:R approximately 1.0. The typical pH of such CO_2_-poor water would be around 11.0–13.0 (refs. ^16,36^). Red and blue shaded areas denote probable pH ranges for waters of icy bodies beyond and within the CO_2_ snowline, respectively^16,36^.

These wider implications are based on the fact that interactions between chondritic rocks and CO_2_-rich fluids at a water:rock (W:R) ratio of around 1.0 would lead to a pH of about 10.0 (refs. ^16,36^), in which dissolved phosphate concentrations tend to maximize^17^ (Fig. 5). This is supported by recent findings for asteroid Ryugu, whose parent body is thought to have formed beyond the CO_2_ snowline^37^. Given the high solubility of Na-bearing phosphate^17^, its common occurrence in Ryugu samples is qualitatively consistent with our conclusion of phosphate-rich waters beyond the CO_2_ snowline. Enceladus’s ocean could be a harbinger of high phosphorus availability in subsurface oceans across most of the outer Solar System. On the other hand, if an icy body formed within the CO_2_ snowline both low [ΣCO_2_] and high pH (approximately 13.0 at W:R around 1.0)^16,36^ would probably limit dissolved phosphate concentrations (Fig. 5).

The stark contrast between earlier modelling and our results might be due to modelling assumptions that were based on scaling fluxes of the P cycle on the modern Earth to Enceladus^15^, not considering the fundamental differences between Earth and ocean-bearing moons. The most important differences are the much higher concentration of carbonate species in alkaline ocean water and the probable presence of unrecycled, equilibrated rocks at the seafloor of Enceladus^34,38^ versus continuous production of more reactive seafloor basalts on Earth. Regardless of these theoretical considerations, with the finding of phosphates the ocean of Enceladus is now known to satisfy what is generally considered to be the strictest requirement of habitability.

## Methods

### CDA data analysis

#### Short description of the CDA chemical analyser subsystem

The chemical analyser is the subsystem of the CDA^10^ that provides compositional information about an impacting dust particle. Depending on the trajectory of the particle, it either hits the central rhodium target (chemical analyser target (CAT), diameter 0.17 m), the surrounding gold target (diameter 0.41 m) or the inner wall of the instrument. This work deals only with impacts on the CAT. If a dust particle impacts the CAT with sufficient energy, it is totally vaporized and partly ionized, forming an impact plasma of target and particle ions together with electrons, neutral molecules and atoms. The instrument separates this plasma, the positive component of which is linearly accelerated towards a multiplier about 19 cm away, used to generate a ToF spectrum. The spectrometer is sensitive to positive ions only. The mass resolution (*m*/Δ*m*), derived from laboratory experiments with the instrument, depends on the atomic masses of the ions. At 1 u, *m*/Δ*m* is 10, increasing to 30 *m*/Δ*m* at 100 u and up to 50 *m*/Δ*m* at 190 u, although these values vary markedly with impact conditions.

Acquisition of a spectrum can be triggered by charge thresholds being exceeded on either the target or the multiplier ion detector (when a certain abundant ion species arrives there, typically H^+^, H_3_O^+^ or Na^+^). In the case of a multiplier-trigger, the spectra show only ions with masses higher than the triggering ion species; the triggering species and those with lower masses do not appear in the spectrum. For this work, all P-rich spectra were triggered by impact. The spectra are logarithmically amplified, digitized at eight-bit resolution and sampled at 100 MHz for a period of 6.4 μs after the trigger. The recording period of the high-rate sampling mode allows the detection of ions with mass of up to 185–200 u, assuming that instrument recording is triggered by the impact itself.

Because ToF is proportional to the square root of the mass:charge ratio of ions, its spectrum in an ideal case also represents a mass spectrum for identical ion charges. The ions created by impact ionization in the impact speed regime considered here (roughly 10 km s^–1^) are almost exclusively singly charged. Unfortunately, ToF is also influenced by the broad distribution of initial ion velocities, slightly varying flight paths and plasma-shielding effects^40^. For that reason, species of identical mass are distributed over a range of sampling points and the mass resolution drops below integer values, usually at 20–30 u and above. Therefore, we prefer to show original CDA spectra with an *x* axis showing ToF rather than mass; the latter could mislead the reader to intuitively assume an unrealistically high accuracy.

For a detailed description of the instrument see ref. ^10^; the calibration routine is described in refs. ^6,20^. For the dataset in this work the mass calibration was generally done using sodium (Na^+^) and sodium hydroxide ((NaOH)Na^+^) mass lines as reference and a stretch factor of *a* = 473 ns.

#### CDA dataset

All data used for this work are listed in Extended Data Table 1 and, like all CDA data, are archived on the Small Bodies Node of the Planetary Data System (PDS–SBN), at https://sbn.psi.edu/pds/resource/cocda.html. We used 15 time periods between 2004 and 2008 covering times of CDA pointing that were favourable for the detection of E-ring dust. Times this early in the Cassini tour were chosen because CDA contamination from salts deposited on the CDA impact target during deep Enceladus plume crossings was then negligible^39^. Within these periods a total of 7,353 spectra from E-ring grains were recorded. A Lee filter was applied to the spectra to improve signal to noise. Of these spectra, 962 have been categorized as Type 3 (Extended Data Table 1).

From this set of Type 3 spectra, nine stand out as belonging to a previously unknown subtype with a unique pattern of three peaks of *m*/*z* above 120 u; these are the P-rich spectra discussed above. Individual events are listed in Extended Data Table 2. Remarkably, all these spectra were triggered on the instrument by the impact charge of the particle whereas only 36% of all Type 3 spectra in the dataset were triggered in this way (Extended Data Table 1). The remaining 64% of Type 3 spectra were triggered by a particular ion species reaching the multiplier (Methods). By applying binomial statistics, we get a probability that all P-rich spectra were impact charge triggered by chance of only 0.01%. Therefore, we assume a systematic effect in spectral recognition for P-rich grains. Indeed, it is plausible that the spectrum of a P-rich grain is not identified as Type 3 if it is triggered by an ion species at the multiplier. Type 3 spectra are in most cases triggered by Na^+^, which is then missing in the spectrum. However, without the Na^+^ line and in the absence of other typical Type 3 features such as (NaCl)_n_Na^+^ or (Na_2_CO_3_)Na^+^, P-rich spectra are not identifiable as being Type 3 following the criteria normally used, which focus on spectra dominated by chloride, carbonate and hydroxide peaks^3,11^.

We therefore used only those 345 spectra of the dataset that—like the new phosphate-rich spectra—have been triggered by impact (Extended Data Table 1) as a reference to calculate the frequency of the new phosphate-bearing subtype within the family of Type 3 grains and, with that, the average phosphate concentration in salt-rich Type 3 grains. It is noteworthy though, that even in the unlikely case that there was no such systematic selection effect and we use the entire dataset for the inference of average phosphate concentration, the lower limit will still be ΣPO_4_^3–^ above 0.3 mM, more than 100-fold the mean concentration in Earth’s oceans, and the conclusions of this work are unaffected.

### Laboratory methods simulating CDA mass spectra

#### Identification of sodium phosphates

The largest peaks in all nine CDA spectra always appear at 23 and 63 u, indicative of Na^+^ and (NaOH)Na^+^ cations that are the defining features in all salt-rich spectra from grains emitted by Enceladus (Type 3; ref. ^3^), thus these spectra contain sodium salts in an alkaline matrix. We used the other three characteristic peaks, at 125, 165 and 187 u (allowing a divergence of ±1 u that reflects the mass uncertainty of CDA^7,10^), to perform a rigorous database search—in both our own database base^41^ as well as several external databases (for example, NIST Chemistry WebBook, MassBank North America and MassBank North Europe (https://massbank.eu/MassBank)). We found phosphates to be the only possible class of Na-rich compounds that produce a pattern that is in even remote agreement with the observed cationic peak pattern. After that we widened the accepted mass range to ±2 u away from the observed masses and relaxed the restriction to Na-rich compounds to determine whether there are other species (without sodium) forming a characteristic mass line pattern similar to the three mass lines (125, 165 and 187 u). The only match remotely close was methyl-2,2-dimethyl-3-(2,2-dichloroethenyl) cyclopropanoate, showing prominent peaks at *m*/*z* 61(−2), 127(+2), 163(−2) and 187. The mass spectrum can be found at ref. ^42^.

It is questionable whether a sufficient concentration of this type of chlorinated organic ester would be stable in Enceladus’s alkaline ocean. Even if we assume that this unlikely substance could be mixed with sodium salts in an ice grain, the spectrum has additional features not matching the CDA spectrum—most prominently a peak at *m*/*z* 91, which is absent in all nine CDA spectra. Similarly, any combination of exotic organic compounds we found that remotely reproduces the five prominent CDA peaks does have a number of additional peaks that are absent in CDA spectra.

In conclusion, sodium phosphates are the simplest and—to the best of our knowledge—the only explanation for all the peaks observed in the CDA spectra (Figs. 1 and 2). These salts have already been predicted to be relatively abundant in Enceladus’s ocean based on recent geochemical modelling^17^, and the observed peak pattern can be readily reproduced in mass spectra from our analogue experiment, as described in the following section.

#### LILBID experiments simulating CDA spectra

We used the laser-induced liquid beam ion desorption (LILBID) facility at Freie Universität Berlin to simulate CDA spectra. The experimental setup is described in detail in ref. ^22^ and here we provide only a brief overview. A micrometre-sized water beam is irradiated by a pulsed infrared laser (20 Hz, 7 ns pulse length) at a wavelength of 2,840 nm and variable laser energy. On absorbing the laser energy, the water beam explosively disperses into neutral and charged macroscopic, molecular and elemental fragments. Cations or anions, depending on the instrument polarity, are accelerated and analysed in a ToF mass spectrometer. Detected signals are amplified, digitized and recorded with a LabVIEW-controlled computer. Typically 300–500 individual spectra are co-added and averaged during one effective measurement. The laboratory setup is calibrated before every measurement using a 10^−6^ M NaCl solution^22^. The recorded mass spectra showed a mass resolution of 600–800 *m*/Δ*m* (full-width at half-maximum).

As discussed in Methods, we found phosphates to be the only Na-rich compounds that are in even remote agreement with the observed cationic peak pattern. Because the appearance of certain peaks and their amplitudes are very sensitive to the phosphate species used and their concentrations, we individually tested a variety of combinations of aqueous solutions of Na_3_PO_4_, Na_2_HPO_4_, NaH_2_PO_4_ and Na_2_HPO_3_, as well as mixtures of these compounds at different concentrations with the goal of reproducing the CDA peak pattern. Concentrations were varied between 0.01 M and about 5 M for NaH_2_PO_4_, 0.5 M for Na_2_HPO_4_ and 1.5 M for Na_3_PO_4_.

We found that a mixture of Na_3_PO_4_ and Na_2_HPO_4_ most closely reproduced the CDA peak pattern of P-rich spectra (Fig. 1), whereas spectra of the pure substances always exhibited differences (Extended Data Figs. 1 and 2).

We also tested for the presence of phosphites (using Na_2_HPO_3_, in which P has an oxidation state of +III) and hypophosphite (using NaH_2_PO_2_, in which P has an oxidation state of +I). Both substances created mass lines not in agreement with CDA observations: (Na_2_HPO_3_)Na^+^ (149 u) for phosphite and (NaH_2_PO_2_)Na^+^ (111 u) for hypophosphite. For phosphite we inferred an upper limit concentration of 5 mM to be in agreement with the absence of a mass line at 149 u in CDA spectra (Extended Data Fig. 3).

The phosphate salts used in our experiments were:

- Na_3_PO_4_: Sigma-Aldrich, 96% purity
- Na_2_HPO_4_: Roth, over 99% purity
- NaH_2_PO_4_•2H_2_O: AppliChem, over 98% purity
- NaH_2_PO_3_•5H_2_O: Sigma-Aldrich, over 98% purity
- NaH_2_PO_2_•H_2_O: Sigma-Aldrich, over 99% purity

#### Theoretical considerations regarding the observed characteristic pattern of phosphate ions

We observed the peak sequence at masses 187, 165 and 125 u as a characteristic pattern indicating the presence of phosphorus (P) salts in Enceladus’s ice grains, present in all nine of the individual CDA ice grain spectra. Phosphate-containing salts from the solvation and extraction of minerals at a basic pH value produced a number of anion species in solution that are coupled through a multistep acid–base thermodynamic equilibrium and proton exchange—for example, PO_4_^3–^ ↔ HPO_4_^2–^ ↔ H_2_PO_4_^–^. For the given high salt concentrations, abundance of counter cations are close to anion abundances in solution (Na_n_PO_4_^(3–n)–^). After ice grain impact, ionic aggregates showed additional clustering with abundant cations such as Na^+^ resulting in characteristic mass peaks due to species including (Na_2_HPO_4_)Na^+^ and (Na_3_PO_4_)Na^+^. Owing to the basic pH of the solution forming the ice grains (or the liquid desorption matrix of the laboratory experiment), these two species are expected.

However, we did not detect a (NaH_2_PO_4_)Na^+^ adduct in CDA spectra but rather a (NaPO_3_)Na^+^ peak at 125 u, a charged molecular aggregate of a metaphosphate with phosphorus in the same oxidation state (+V) as the other orthophosphates. We attribute the absence of (NaH_2_PO_4_)Na^+^ and the appearance of (NaPO_3_)Na^+^ instead to an elimination reaction during desorption (LILBID) or particle impact (CDA):

NaH_2_PO_4_ + heat/energy → NaPO_3_ + H_2_O, followed by the addition of Na^+^.

The fact that the (NaPO_3_)Na^+^ peak at 125 u is formed from hydrogen phosphates (linked via an acid–base equilibrium) in our analogue experiments, but not from Na_3_PO_4_ alone, supports this picture. However, it should be noted that we could produce a (NaH_2_PO_4_)Na^+^ peak (143 u) if high concentrations (over around 0.05 M) of NaH_2_PO_4_ or Na_2_HPO_4_ (Extended Data Fig. 2) were used in the laboratory experiments or if the pH value of the solution was forced below about 9.0. We then find a clear correlation of the amplitudes of dihydrogen phosphate (143 u) with metaphosphate (125 u). In the CDA data the 143 u peak appears to be below the noise level of the detector. In our laboratory spectra the 125 u metaphosphate peak is generally three- to fivefold more intense and it is therefore likely that small amounts of (NaH_2_PO_4_)Na^+^ (143 u) were buried in the noise of the CDA spectrum. Such a signature was indeed tentatively observed in one of the nine spectra (event 3 in Extended Data Table 1).

**Extended Data Fig. 4:**
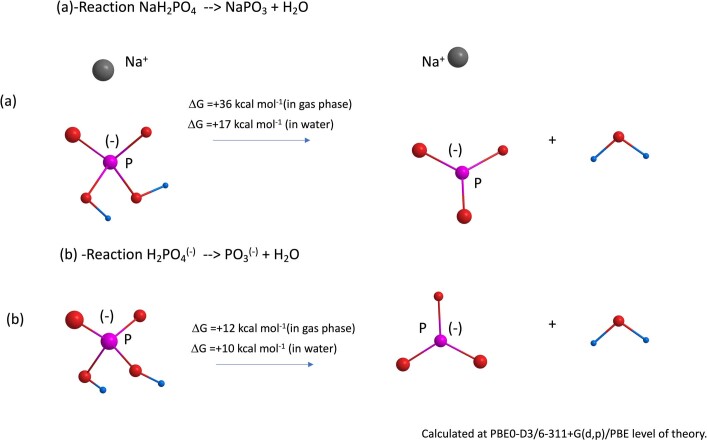
Jaguar quantum chemistry program package at the PBE0-D3/6-311+G(d,p)/PBE level of theory of the elimination reaction from dihydrogen phosphate leading to the observed meta phosphate species. The presence of water significantly reduces the meta phosphate formation barrier, specifically in the case with Na^+^.

To test these ideas we calculated the thermochemical properties of the water elimination of NaH_2_PO_4_ + heat/energy → NaPO_3_ + H_2_O with the Jaguar quantum chemistry programme package at the PBE0-D3/6-311+G(d,p)/PBE level of theory. Two variants have been calculated: (1) with Na^+^ as a counter ion and (2) without Na^+^ (involving a H_2_PO_4_^–^ anion). The reaction is slightly endergonic in the gas phase and even more favoured in/with water. The barrier of the concerted elimination reaction appears to be low if water molecules are involved. One may even consider it to be a water-assisted reaction, in particular for the case of Na^+^ as counter ion (Extended Data Fig. 4).

### Hydrothermal experiments and calculations of chemical equilibria

#### Experimental methods and procedures

Hydrothermal experiments were performed with a Dickson-type hydrothermal apparatus (Toyo Koatsu Co. Ltd) used in a previous study^43^. In this apparatus, water–rock reactions occur in a flexible reaction cell that comprises a gold bag with a titanium head. The flexible reaction cell was connected to a stainless steel sampling tube with a gold-coated inner wall, to avoid catalytic reactions. The reaction cell was heated at 500 °C for 3 h in air before each experiment to remove potential contamination by organic matter. The reaction cell was placed in a stainless steel autoclave, which was held at a pressure and temperature of 30 MPa and 150 °C, respectively, during the experiments. The reaction cell was pressurized by the addition of surrounding water into the autoclave using a hand pump. The autoclave was heated with an external heater. Due to the flexibility of the gold bag, the reaction cell deforms plastically without fracturing in response to the addition of surrounding water. This allowed us to undertake online sampling of fluid without significant changes in pressure.

The starting rock material was prepared by powdering a few pieces of the commercially available Jbilet Winselwan carbonaceous chondrite (CM-2 type)^25^ in an agate mortar. The starting solutions were prepared by dissolving NaHCO_3_ powder and NH_3_ solution in ultrapure water such that the initial ΣCO_2_ was 0.2 M for Run no. 1 and 1.0 M for Run no. 2, and ΣNH_3_ was 0.5 M. One way to achieve high ΣCO_2_ in an Enceladus experiment is to introduce gaseous CO_2_ in the reaction cell and dissolve it by pressurization. However, this is technically challenging in our experimental system and we cannot control the [ΣCO_2_] and pH of the starting fluids. Therefore, we achieved high ΣCO_2_ by the addition of NaHCO_3_ powder. We added 1.0 M Na in Run no. 2, higher than Na concentrations in Enceladus’s ocean^3^. If Na phosphate (for example, Na struvite) had precipitated in such Na-rich fluids of Run no. 2, this would have affected the buffer system that controls ΣPO_4_ in fluids. Although the temperature and pressure dependences of the thermodynamic constants of Na struvite have not been experimentally determined, the saturation index (log([Na^+^][Mg^2+^][HPO_4_^2–^]/*K*_Na-struvite_), where *K*_Na-struvite_ denotes the equilibrium constant from ref. ^17^ of Na struvite at 1 bar and 25 °C, is calculated to be below −1.6 using the measured Na, Mg and ΣPO_4_ concentrations and in situ pH. Because the fluid Mg concentrations of Run no. 2 were below the detection limit (Extended Data Fig. 8b), Na struvite was likely to have been undersaturated in the experiment. The presence of Ca sulfate in the starting rock materials (CM chondrite) also does not affect our conclusion regarding the hydroxyapatite-calcite buffer system. If the hydroxyapatite-gypsum (or hydroxyapatite-anhydrite) buffer controls ΣPO_4_^3–^, [ΣPO_4_^3–^] should dramatically increase with [SO_4_^2–^]; however, this did not occur (Extended Data Table 3). The initial amounts of starting rock powder and solution were around 7 g and 10 ml, respectively. In each experiment we collected several aliquots (about 1.2 ml) of fluid for analysis. As such, water:rock mass ratios in the reaction cell changed from around 1.4–0.9 due to the removal of multiple fluid samples.

The collected fluid samples were analysed by inductively coupled plasma atomic emission spectroscopy (ICP–AES: SPS5510, Hitachi High-Tech) for dissolved Na, K, Mg, Ca, Si and Al content; by ICP mass spectrometry (ICP–MS: Agilent 8000) for dissolved Fe content; by ion chromatography (ICS-1600, DIONEX) for dissolved Cl and SO_4_ content; and by gas chromatography (GC-2010, Shimadzu) for ΣCO_2_ content (Extended Data Table 3). ΣPO_4_^3–^ content was analysed by both tandem ICP–MS (ICP–MS/MS: Agilent 8000, Agilent) and ion chromatography. The former methodology quantified dissolved total P in fluids at high sensitivity, including phosphate and potentially phosphite, whereas the latter directly measured ΣPO_4_^3–^ with moderate sensitivity. The results of the two analyses showed that, basically, all dissolved total P in the fluids was phosphate (Extended Data Table 3). As such, we used ICP–MS/MS results to quantify ΣPO_4_^3–^ in the fluid samples. In situ pH values at 150 °C during the experiments were calculated using the geochemical code PHREEQC v.3 (ref. ^44^), based on the measured pH at 25 °C and dissolved elements, molecules (phosphate and sulfate) and ΣCO_2_ in the diluted fluid samples. To calculate the in situ pH of the alkaline, Na-carbonate-rich fluids, we constrained the charge balance from the measured pH at 25 °C in two ways. First, Na was used to compensate for charge imbalance due to analytical uncertainties. Second, ΣCO_2_ was used to compensate for charge imbalance. We adopted the median value between the two values as the in situ pH, and the difference between the median and upper/lower values as the error. We also conducted mineralogical and chemical analyses of solid residues after the experiments using an X-ray diffraction spectrometer (XRD: MiniFlex600, Rigaku) and a scanning electron microscope with an electron probe microanalyser (EPMA: JXA-8530F, JEOL).

The ΣPO_4_^3–^ concentrations controlled by the hydroxyapatite-calcite and whitlockite-calcite buffer systems were calculated with the equilibrium constants obtained from the SUPCRT92 programme^45^. In the calculations, the thermodynamic properties of whitlockite (β-Ca_3_(PO_4_)_2_) and hydroxyapatite were taken from Robie et al.^46^.

#### Elemental composition of carbonate and phosphate

**Extended Data Fig. 5:**
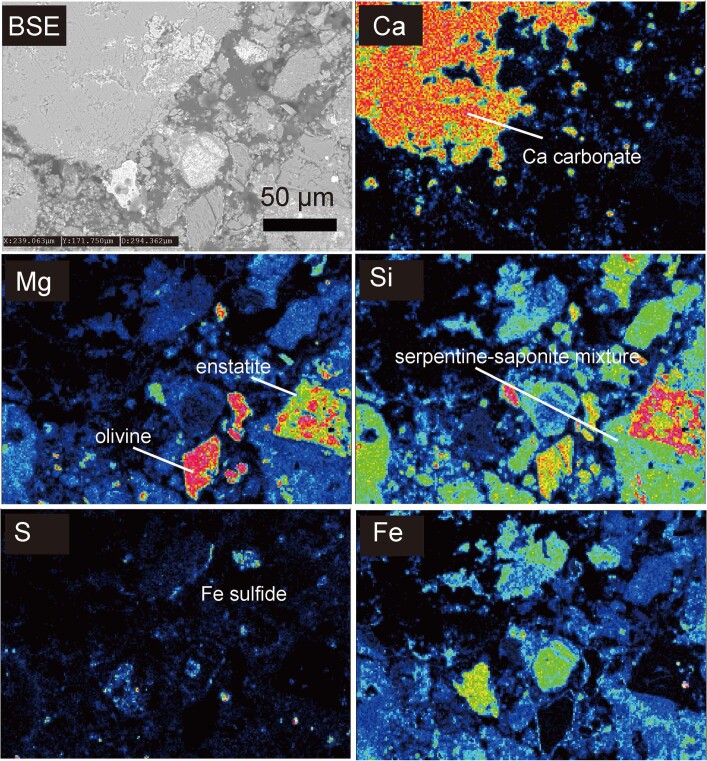
Typical Backscattered Electrons (BSE) SEM images and elemental mapping for solid residues collected after experiment Run #1. The presence of large Ca carbonate grains (>100 μm) indicates the formation of calcite during the experiment. Such large Ca carbonate grains were absent from the sample before our experiments (Extended Data Fig. 7). XRD analysis (Extended Data Fig. 10) shows that the precipitated Ca carbonates are most likely calcite.

**Extended Data Fig. 6:**
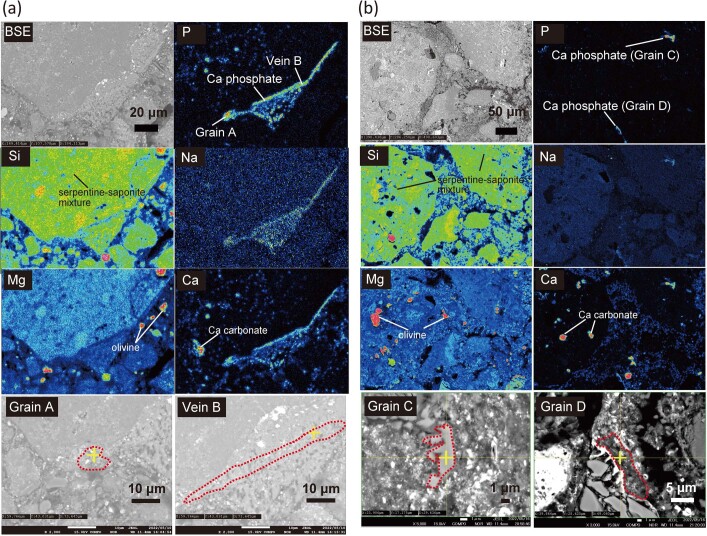
**(a)** Typical Backscattered Electrons (BSE) SEM images and elemental mapping results for Ca phosphate minerals in the solid residues collected after experiment Run #1. Phosphate-rich grains with a size of several micrometres and veins with a width of several micrometres and length of ~100 μm are seen near a large serpentine grain. In addition to Ca and P, Na is enriched in Ca phosphate minerals. Close-up images of phosphate minerals (the bottom two images) indicate that Ca phosphate coexists with other minerals (e.g., serpentine and saponite). **(b)** Same as Extended Data Fig. 6a, but for experiment Run #2. Phosphate-rich grains with a size of several micrometres are observed (see the bottom two SEM images for close-up views of phosphate minerals). Close-up images of Grain D indicate that Ca phosphate minerals coexist with other minerals, whereas the surface of Grain C exhibits relatively uniform composition. The chemical compositions of Ca phosphate (Grain A and Vein B) revealed by microprobe spot analysis are summarized in Extended Data Table 4. XRD analysis (Fig. 10) shows that the precipitated Ca carbonates are most likely calcite.

**Extended Data Fig. 7:**
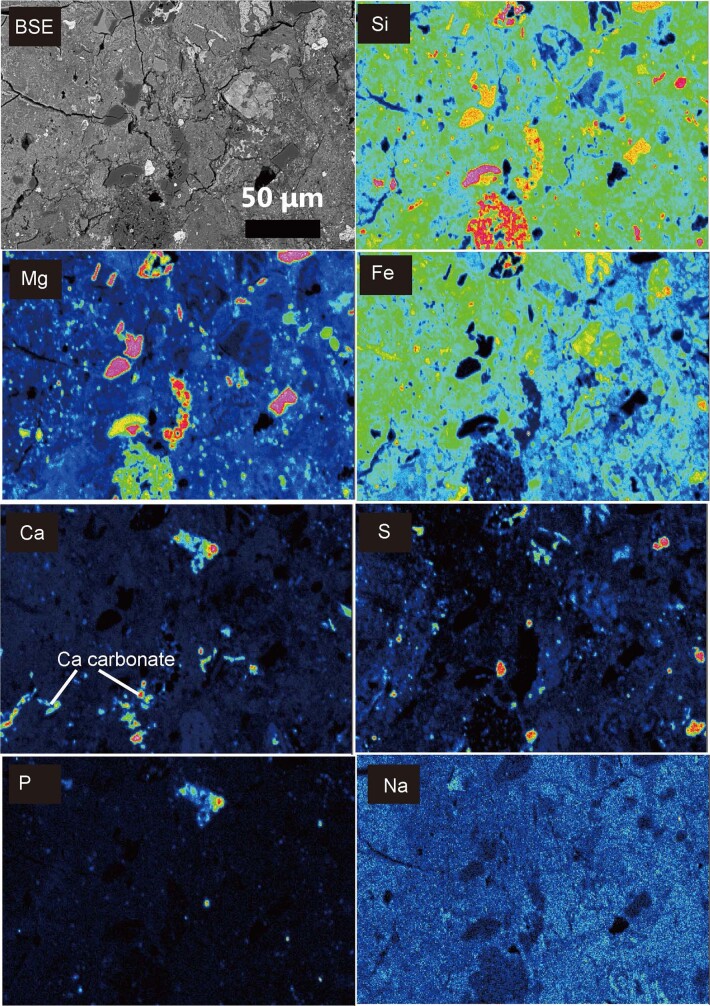
Typical SEM images and elemental mapping results of carbonaceous chondrite samples before the experiments. The size of Ca carbonate grains is typically ~10 μm or less.

**Extended Data Fig. 8:**
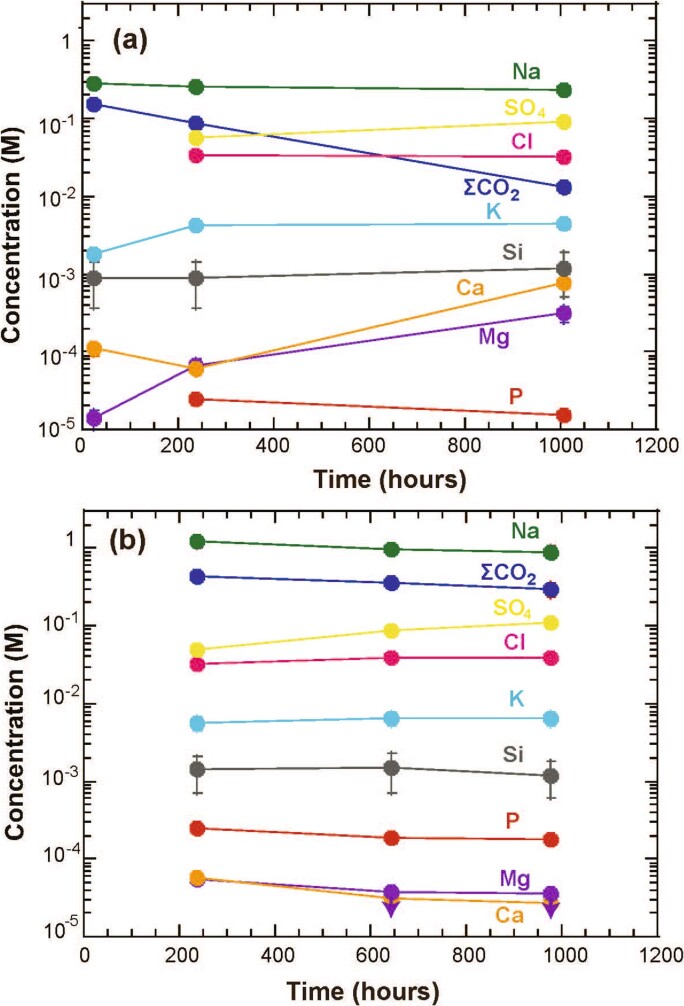
Temporal variations in the concentrations of selected elements (Na, K, Si, Mg, Ca, and P) and dissolved total inorganic carbon (ΣCO_2_). Results for (a) Run #1 and (b) Run #2 are shown. The arrows for Mg concentrations at 650 and 1000 h of reaction time for Run #2 indicate that the concentrations were less than the detection limit (0.036 mM). The measured data and calculated in situ pH of fluids are summarized in Extended Data Table 3. The decreases in ΣCO_2_ in both experiments over time were caused by the formation of carbonate minerals (i.e., calcite), as confirmed by microprobe and the results of XRD analysis shown in Extended Data Figs. 5 and 10, respectively. Where no error bars are shown, the analytical errors are smaller than the data points. All measured data, analytical errors, and calculated in situ pH of fluids are summarized in Extended Data Table 3.

Extended Data Figs. 5 and 6 show typical secondary electron microprobe images and elemental mapping for thick sections of solid residues after the experiments (Run nos. 1 and 2). In the solid residues we found large grains of precipitated Ca carbonate (CaCO_3_, size over 100 μm; Extended Data Fig. 5 and Extended Data Table 4). Because the typical size of Ca carbonate in the sample before the experiments was 10 μm or less (Extended Data Fig. 7), the large Ca carbonate grains found after the experiments are highly likely to have been precipitated during the experiments through ΣCO_2_ sequestration. This view is supported by decreases in ΣCO_2_ in fluids (Extended Data Fig. 8). Our XRD analysis shows that the precipitated Ca carbonates are most likely calcite in both of the experiments (Run nos. 1 and 2; Extended Data Fig. 10).

**Extended Data Fig. 9:**
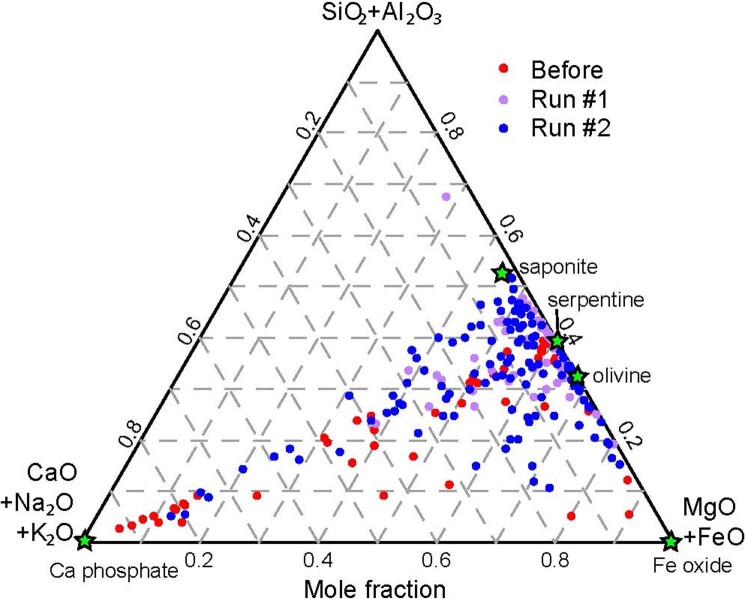
Ternary diagram of the results of EPMA analysis of the chemical compositions of phyllosilicate and Ca phosphate-rich grains in the samples before and after the experiments, plotted as molar fractions. Green stars indicate the compositions of pure minerals found in the samples.

To investigate the composition of the phyllosilicate phase before and after the experiments, we performed EPMA analysis for the phyllosilicate matrix around Ca phosphate grains. Extended Data Fig. 9 shows a ternary diagram with the results of EPMA analysis for phyllosilicate, Ca phosphate and Fe oxides in the samples before and after the experiments. Phyllosilicates in the starting materials were mostly serpentine; however, mixtures of serpentine and saponite appeared in the samples after the experiments. Extended Data Fig. 9 indicates a linear trend of a mixing line of the compositions between Ca phosphate and serpentine (and saponite mixtures).

Extended Data Table 4 presents the results of spot analysis for Ca phosphate and carbonate found in the solid residues after the experiments of Run nos. 1 and 2. At the micrometre scale, Ca phosphate minerals usually coexist with other secondary minerals such as serpentine and saponite (Extended Data Fig. 6), and thus the results of the spot analysis might not be explained by the simple chemical composition of an alteration mineral. As reported in Extended Data Table 4, Grain C with the highest P content had high total mass fractions of around 95–100 wt%, suggesting that the contribution of calcite to Ca:P atomic ratios is small (less than 2–3% of Ca mole abundance), whereas serpentine and saponite would coexist with Ca phosphate of Grain A, Vein B and Grain D. These hydrated secondary minerals can reduce the total mass fractions of spot analyses by 5–10% owing to the presence of H_2_O and OH. Because the total mass fractions for Grain A, Vein B and Grain D are also high in general (over 87 wt%; Extended Data Table 4), calcite is presumed to be rare in Ca phosphate grains, suggesting that the contribution of calcite to Ca:P ratios is small.

**Extended Data Fig. 10:**
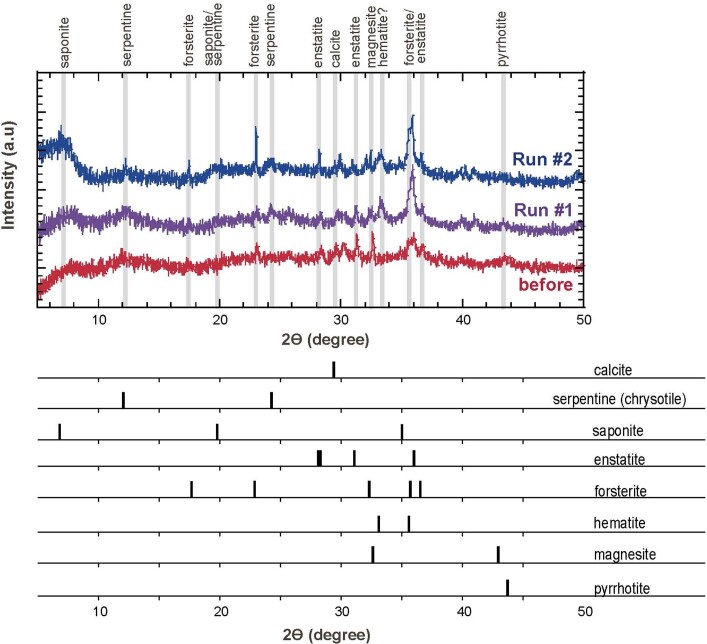
Comparison of X-ray diffraction (XRD) spectra of the solid samples before the experiments (red), after experiment Run #1 (purple), and after experiment Run #2 (blue). a.u., arbitrary units. The XRD intensities are offset for clarity. The primary XRD peaks of minerals that correspond to the XRD peaks are also indicated.

Grain C of Run no. 2 has very high P:Si ratios (Extended Data Table 4c). The measured Ca:P mole ratios are 1.32–1.37. Grain D of Run no. 2 has relatively high P:Si ratios, above 1.0 (Extended Data Table 4d). The Ca:P mole ratios of Grain D range from 1.29 to 1.34. The Ca:P ratios of hydroxyapatite, whitlockite [β-Ca_3_(PO_4_)_2_] and Mg-whitlockite [Ca_9_MgH(PO_4_)_7_] are 1.67, 1.50 and 1.29, respectively. Ca phosphates collected after Run no. 2 (Grains C and D) are inferred to consist of mixtures of multiple Ca phosphates. Due to the trace amounts of Ca phosphates in the solid residues, these could not be identified in the XRD spectra (Extended Data Fig. 10)

In Ca phosphate grains of Run no. 1 (Grain A and Vein B), P:Si mole ratios are generally low (roughly 0.1–1.0) (Extended Data Table 4a,b), suggesting that Ca phosphates coexist with serpentine and saponite. There is a relatively wide variation in the Ca:P mole ratios of Grain A and Vein B, from approximately 1.1 to 1.5 (Extended Data Table 4a,b). A high Ca:P ratio of around 1.5 requires the presence of hydroxyapatite and/or whitlockite. To explain the low Ca:P ratios (below 1.29) of the analysed spots on Grain A and Vein B, the presence of Ca-poor phosphate, such as NH_4_MgPO_4_, would be needed.

Our results showing high C:P ratios (1.32–1.37) for Run no. 2 and low Ca:P ratios (1.25–1.27) for Run no. 1 are consistent with our interpretation of the buffer system that controls ΣPO_4_^3−^ concentrations in the fluid samples. The Ca phosphate grains (C and D) of Run no. 2 contain high fractions of phosphate with high Ca:P, such as hydroxyapatite. This observation is consistent with the control of ΣPO_4_^3−^ concentrations in fluid samples of Run no. 2 by the hydroxyapatite-calcite buffer (Fig. 3). In contrast, the observation of Ca phosphate grains with low Ca:P for Run no. 1 (for fluid with ΣCO_2_ = 0.01 M and reaction time 1,000 h) is consistent with the control of ΣPO_4_^3−^ concentrations by Ca phosphate with lower Ca:P ratios, such as whitlockite, rather than control by hydroxyapatite (Fig. 3). This fluid sample was collected in the final sampling of Run no. 1 (Extended Data Fig. 8a), and the solid residue was collected after this final sampling. We consider that Ca phosphate with relatively low Ca:P, such as whitlockite [β-Ca_3_(PO_4_)_2_], Mg-whitlockite and NaMgPO_4_, would have been precipitated before the final sampling in response to an increase in Mg concentration in the fluid (Extended Data Fig. 8), and that the whitlockite-calcite buffer would have controlled the P concentration of the fluid at the end of experimental Run no. 1.

#### Chemical equilibrium considerations regarding phosphate buffering systems

**Extended Data Fig. 11:**
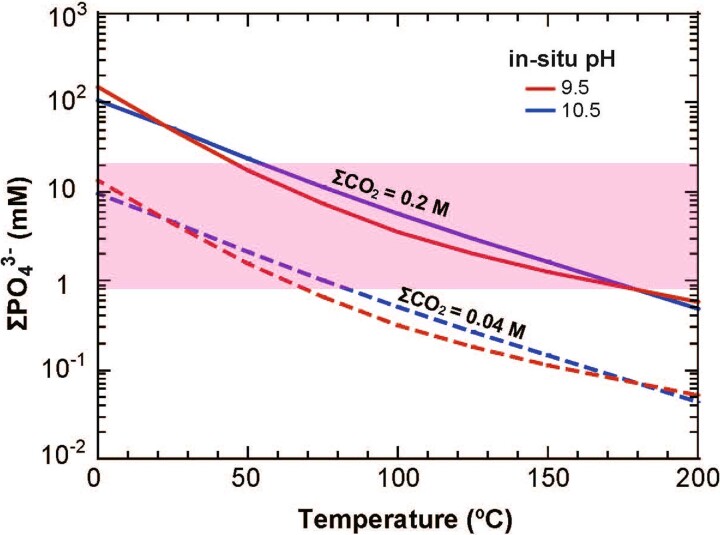
Results of calculated ΣPO_4_^3−^ in fluids as a function of temperature for different pH and ΣCO_2_ assuming the whitlockite–calcite buffer system. Pink shading represents the observationally-derived range of ΣPO_4_^3−^ from the present study.

Extended Data Fig. 11 shows the calculated results of [ΣPO_4_^3−^] in fluids as a function of temperature for different pH and [ΣCO_2_] values, assuming the whitlockite-calcite buffer system (see above for the equation). Although our experimental data suggest that the whitlockite-calcite buffer could have controlled ΣPO_4_^3−^ concentrations in the fluid at the end of Run no. 1, we consider that this could have been an experimental artefact reflecting transient states of supersaturation of Mg-bearing phases. In the experiments, the dissolution of anhydrous primordial minerals (for example, olivine and pyroxene) would have provided Mg and Ca for the fluids. These Mg and Ca ions would have been removed by reactions with CO_3_^2−^, forming carbonate minerals (Extended Data Fig. 5). However, at the end of Run no. 1, ΣCO_2_ in the fluids was depleted (Extended Data Fig. 8) owing to CO_2_ sequestration via carbonate mineral formation; therefore, dissolved Mg and Ca concentrations increased in the fluids (Extended Data Fig. 8). In longer-period reactions, excess Mg and Ca ions would have been consumed through the formation of serpentine and saponite. Thermodynamic equilibrium calculations show that Mg and Ca concentrations in fluids that interact with carbonaceous chondritic rocks are typically low (0.001–0.010 mM each) in alkaline fluids (pH over 9.0) in the presence of serpentine and saponite^36,47^. In Enceladus, water–rock reactions would be driven closer to acid–base equilibrium because the reactions can occur over much longer time scales than apply in our experiments. If the ocean of Enceladus were consistent with the expectation of low Mg and Ca concentrations (for example, under 0.1 mM each), then ΣPO_4_^3−^ in fluids in contact with chondritic rocks would be controlled by the hydroxyapatite-calcite buffer system.

In this study, we considered hydroxyapatite and whitlockite [β-Ca_3_(PO_4_)_2_] as phosphate minerals of buffer systems that control [ΣPO_4_^3−^] in fluids. However, previous work has suggested the possibility of the occurrence of other phosphate minerals in water–rock reactions in Enceladus^17^, including Mg-whitlockite and merrillite. Using the equilibrium constants of dissolution of Mg-whitlockite and merrillite at 1 bar and 25 °C (ref. ^48^), and assuming [Mg^2+^] as roughly 10^−5^ M in the fluid samples (Extended Data Table 3), ΣPO_4_^3−^ values controlled by the Mg-whitlockite-calcite and merrillite-calcite buffer systems (that is, 6 × 10^−5^ and 7 × 10^−6^ M, respectively, for ΣCO_2_ = 0.1 M) are about 1/30 to 1/250 that of the hydroxyapatite-calcite buffer (that is, 2 × 10^−3^ M for ΣCO_2_ = 0.1 M). Although the temperature and pressure dependences of the thermodynamic equilibrium constants of both Mg-whitlockite and merrillite are unknown, the observed ΣPO_4_^3−^ values in our experiments are unlikely to be explained by these buffer systems.

We also assumed that calcite is the carbonate mineral phase for the buffer system, but dolomite is usually more abundant than calcite in CI chondrites^49^. However, ΣPO_4_^3–^ values controlled by the hydroxyapatite-dolomite buffer are orders of magnitude higher than those controlled by the hydroxyapatite-calcite buffer for ΣCO_2_ = 0.1 M and the typical Mg concentration in our experiments ([Mg^2+^] = 10 M^–5^). Therefore, we conclude also that the hydroxyapatite-dolomite buffer may not explain the observed ΣPO_4_^3−^ in our experiments.

## Online content

Any methods, additional references, Nature Portfolio reporting summaries, source data, extended data, supplementary information, acknowledgements, peer review information; details of author contributions and competing interests; and statements of data and code availability are available at 10.1038/s41586-023-05987-9.

## Figures and Tables

**Extended Data Table 1:**
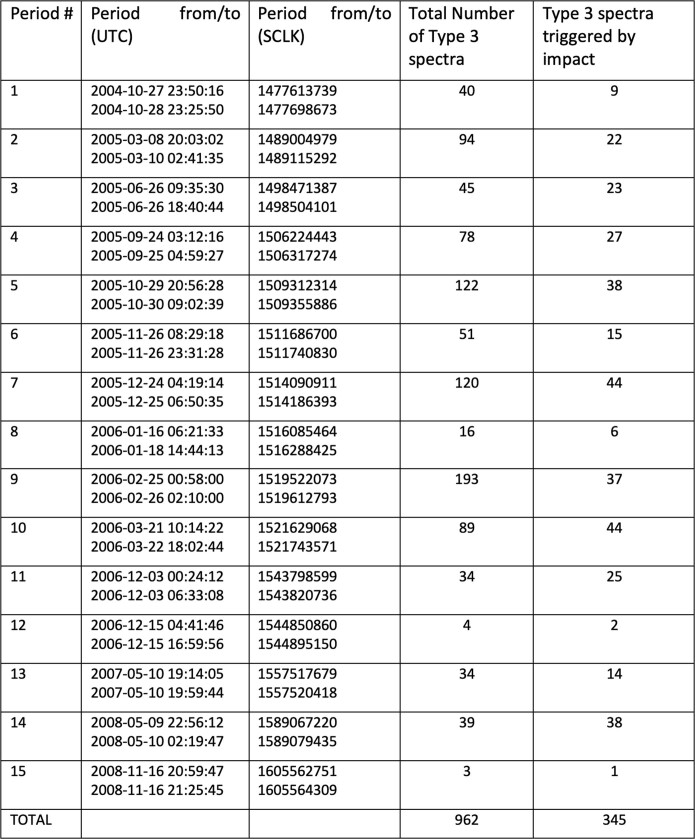
CDA set of Type 3 spectra used for this work

**Extended Data Table 2:**
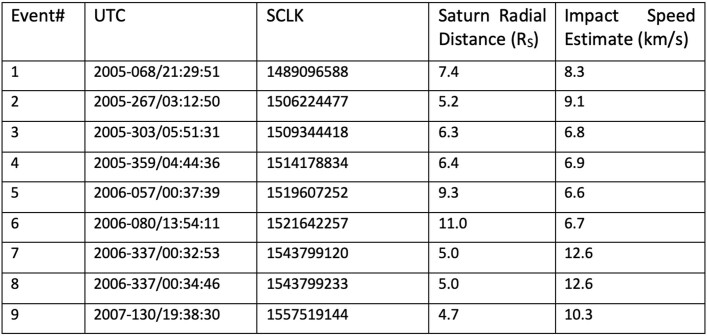
Events of phosphate-rich ice grain recorded by CDA. Saturn distance is given in Saturn radii (equatorial radius R_S_ = 60268 km)

Since the impact speed onto CDA cannot be measured directly for these grains, the relative velocity of the spacecraft to a circular Keplerian orbit provides a rough estimate.

**Extended Data Table 3:**
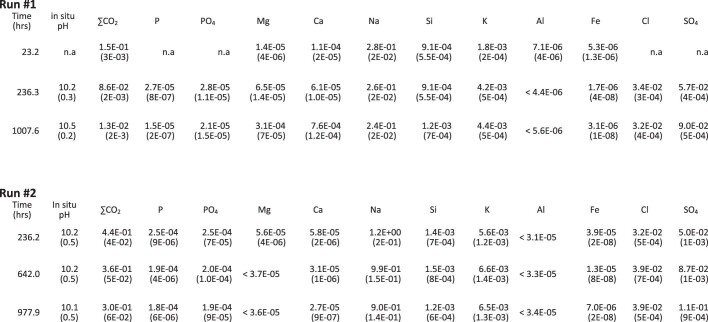
Temporal variations in dissolved species in fluids (in M), with pH at the calculated in-situ pH at 150 °C and 30 MPa

Analytical errors are shown in parentheses. For P concentrations, the values of “P” are the results of total dissolved P in fluids measured using a tandem inductively coupled plasma mass spectrometer (ICP–MS/MS); and those of “PO_4_” are the results of ΣPO_4_^3−^ measured using ion chromatography (see Methods 3a). In cases where element concentrations were too low to be measured (i.e., Mg and Al), the upper limits of concentration (detection limits) are given.

**Extended Data Table 4:**
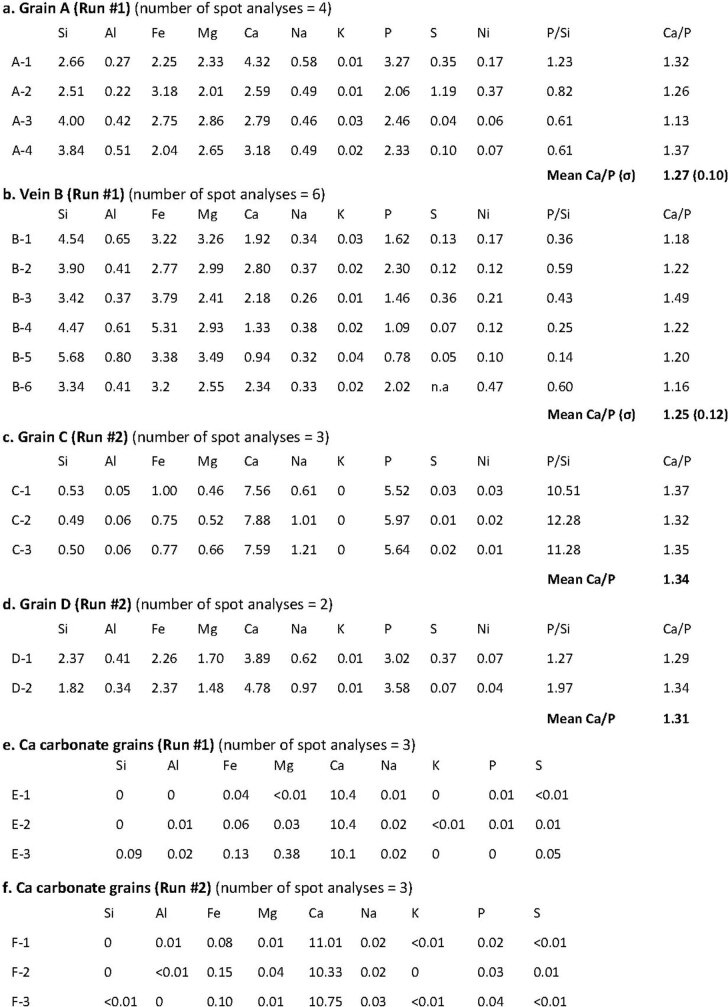
Results (mole abundance in mol/kg) of electron probe microanalyzer (EPMA) analyses for Ca phosphates [Grain A and Vein B for Run #1, and Grains C and D for Run #2 (see Extended Data Fig. 6)], and for Ca carbonate minerals for Runs #1 and #2

The mass fractions of Fe and S were calculated assuming their forms of FeO and SO_3_.

## Data Availability

All CDA data used for this work are listed in Extended Data Table 1 and are archived on PDS–SBN, at https://sbn.psi.edu/pds/resource/cocda.html. Data from the LILBID analogue experiment reproducing CDA data and geochemical experiments are available in the Zenodo public repository at 10.5281/zenodo.7703848.
